# Supplementary material for: Structural basis for effector transmembrane domain recognition by type VI secretion system chaperones
Source: eLife. 2020 Dec 15;9:e62816. doi: 10.7554/eLife.62816 (PMC7773334; doi:10.7554/eLife.62816)
Supplement: Supplementary file 4. [file elife-62816-supp4.docx]

**Supplementary File 4: Strains used in this study.**

| Organism | Genotype | Description | Reference |
| --- | --- | --- | --- |
| *P. protegens* Pf-5 | wild-type |  | (Paulsen et al., 2005) |
|  | ΔPFL_6095 | *eagR1* deletion strain | This study |
|  | ΔPFL_6099 | *eagT2* deletion strain | This study |
|  | ΔPFL_6209 | *tne2* deletion strain | (Tang et al., 2018) |
|  | ΔPFL_6096 | *rhsA* deletion strain | (Tang et al., 2018) |
|  | ΔPFL_6079 | *pppA* deletion strain | This study |
|  | ΔPFL_6094 | *vgrG1* deletion strain | This study |
|  | ΔPFL_6096 ΔPFL_6097 attB::*lacZ*, Tet^R^ | *rhsA rhsI* deletion strain, constitutive lacZ expression, Tet^R^ | This study |
|  | ΔPFL_6079 ΔPFL_6096 ΔPFL_6097 attB::*lacZ*, Tet^R^ | *pppA rhsA rhsI* deletion strain, constitutive lacZ expression, Tet^R^ | This study |
|  | ΔPFL_6079 ΔPFL_6209 ΔPFL_6210 attB::*lacZ*, Tet^R^ | *pppA tne2 tni2* deletion strain, constitutive lacZ expression, Tet^R^ | This study |
|  | His_10_-PFL_6096 | Expresses RhsA with a N-terminal His_10_ tag | This study |
|  | ΔPFL_6095 His_10_-PFL_6096 | *eagR1* deletion strain expressing His_10_-RhsA | This study |
|  | ΔPFL_6099 His_10_-PFL_6096 | *eagT2* deletion strain expressing His_10_-RhsA | This study |
|  | His_10_-PFL_6209-VSV-G | Expresses Tne2 with a N-terminal His_10_ tag and a C-terminal VSV-G tag | This study |
|  | ΔPFL_6095 His_10_-PFL_6209-VSV-G | *eagR1* deletion strain expressing His_10_-Tne2-VSV-G | This study |
|  | ΔPFL_6099 His_10_-PFL_6209-VSV-G | *eagT2* deletion strain expressing His_10_-Tne2-VSV-G | This study |
|  | ΔPFL_6095 His_10_-PFL_6096_Δ2-74 | *eagR1* deletion strain expressing His_10_-RhsA lacking its N-terminal TM region | This study |
|  | ΔPFL_6081 | *tssM* deletion strain | (Tang et al., 2018) |
|  | PFL_6096_Δ2-74 | Expresses RhsA lacking its N-terminal TM region | This study |
|  | FLAG-PFL_6094 His_10_-PFL_6096 | Expresses VgrG1 with a N-terminal FLAG tag and His_10_-RhsA | This study |
|  | FLAG-PFL_6094 His_10_-PFL_6096_∆2-74 | Expresses VgrG1 with a N-terminal FLAG tag and His_10_-RhsA_∆NT_ | This study |
|  |  |  |  |
| *P. aeruginosa* PAO1 | ΔPA4856 | *retS* deletion strain | (Goodman et al., 2004) |
|  | ΔPA4856 ΔPA0091 | *retS vgrG1a* deletion strain | (Whitney et al., 2014) |
|  | ΔPA4856 ΔPA0093 | *retS tse6* deletion strains | (Whitney et al., 2014) |
|  | ΔPA4856 ΔPA0094 | *retS eagT6* deletion strain | (Whitney et al., 2015) |
|  | ΔPA4856 PA0093_D11A | *retS* deletion strain expressing Tse6^D11A^ | This study |
|  | ΔPA4856 PA0093_H15A | *retS* deletion strain expressing Tse6^H15A^ | This study |
|  | ΔPA4856 PA0093_D11A_H15A | *retS* deletion strain expressing Tse6^D11A, H15A^ | This study |
|  | ΔPA4856 PA0093_∆2-61, ∆180-222 | *retS* deletion strain expressing Tse6^∆prePAAR, ∆TMDs^ | (Quentin et al., 2018) |
|  | ΔPA4856 PA0093_∆16-61, ∆180-222 | *retS* deletion strain expressing Tse6^∆TMDs^ | This study |
| *E. coli* SM10 λpir | *thi thr leu tonA lac Y supE recA*::RP4-2-Tc::Mu | Conjugation strain | BioMedal LifeScience |
| *E. coli* XL-1 Blue | *recA1* *endA1* *gyrA96 thi-1 hsdR17 supE44 relA1 lac* [F´ *proAB lacI*^q^ Z∆*M15* Tn*10* (Tet^R^)] | Cloning strain | Novagen |
| *E. coli* BL21 (DE3) CodonPlus | F^-^ ompT gal dcm lon hsdS_B_(r_B_^-^ m_B_^-^) λ(DE3) pLysS(cm^R^) | Protein expression strain | Novagen |
