# Supplementary material for: Structural basis for effector transmembrane domain recognition by type VI secretion system chaperones
Source: eLife. 2020 Dec 15;9:e62816. doi: 10.7554/eLife.62816 (PMC7773334; doi:10.7554/eLife.62816)
Supplement: Supplementary file 5. [file elife-62816-supp5.docx]

**Supplementary File 5: Plasmids used in this study.**

| Plasmid | Relevant features | Reference |
| --- | --- | --- |
| pETDuet-1 | Co-expression vector with *lacI*, T7 promoter, N-terminal His_6_ tag in MCS-1, Amp^R^ | Novagen |
| pRSETA | Expression vector with *lacI*, T7 promoter, N-terminal His_6_ tag and a HRV 3C protease cleavage site, Amp^R^ | Life Technologies |
| pET29b | Expression vector with *lacI*, T7 promoter, C-terminal His_6_ tag, Kan^R^ | (Rietsch et al., 2005) |
| pEXG2 | Allelic replacement vector containing *sacB*, Gm^R^ | (Baynham et al., 2006) |
| pSW196 | MiniCTX1 plasmid, Tet^R^ | (Mougous et al., 2006) |
| pSCrhaB2-CV | Expression vector with *PrhaB*, Tmp^R^ | (Cardona and Valvano, 2005) |
| pPSV39-CV | Expression vector with *lacI*, *lacUV5* promoter, C-terminal VSV-G tag, Gm^R^ | This study |
| pSW196::*lacZ* | *lacZ* in miniCTX1 plasmid | This study |
| pETDuet-1::His_6_-ECL_01567-FLAG ::ECL_01568 | Co-expression vector for N-terminal His_6_ and C-terminal FLAG tagged RhsA and RhsI from *E. cloacae* | This study |
| pETDuet-1::His_6_-SF0266-FLAG | Expression vector for class I prePAAR effector SF0266 from *S. flexneri* | This study |
| pETDuet-1::His_6_-SL1344_0286-FLAG :: SL1344_0286a | Co-expression vector for N-terminal His_6_ and C-terminal FLAG tagged Rhs1 and untagged RhsI from *S.* Typhimurium | This study |
| pETDuet-1::His_6_-PA0093-FLAG ::PA0092 | Co-expression vector for N-terminal His_6_ and C-terminal FLAG tagged Tse6 and Tsi6 from *P. aeruginosa* | This study |
| pETDuet-1::His_6_-Spro_3017_FLAG::Spro_3018 | Co-expression vector for N-terminal His_6_ and C-terminal FLAG tagged Tre1 and Tri1 from *S. proteamaculans* | This study |
| pETDuet-1::His_6_-PFL_6096::PFL_6097 | Co-expression vector for N-terminal His_6_- tagged RhsA and RhsI from *P. protegens* | This study |
| pETDuet-1::His_6_-PFL_6096_∆2-74::PFL_6097 | Co-expression vector for N-terminal His_6_-tagged RhsA_∆NT_ and RhsI from *P. protegens* | This study |
| pETDuet-1::PA0093_1-61-His_6_::PA0094-VSV-G | Co-expression vector for C-terminal His_6_ tagged Tse6 TMD1 and C-terminal VSV-G tagged EagT6 | This study |
| pETDuet-1:: SL1344_0286_1-59-His_6_:: SL1344_0285-VSV-G | Co-expression vector for C-terminal His_6_ tagged Rhs1 TMD1 and C-terminal VSV-G tagged SciW | (Vance et al., 2005) |
| pETDuet-1::His_6_-PFL_6209::PFL_6210 | Co-expression vector for N-terminal His_6_-tagged Tne2 and Tni2 from *P. protegens* | This study |
| pETDuet-1::His_6_- SL1344_0286_∆1-59-FLAG :: SL1344_0286a | Co-expression vector for N-terminal His_6_ and C-terminal FLAG tagged Rhs1_∆NT_ and RhsI from *S.* Typhimurium | This study |
| pETDuet-1::His_6_-PA0093::PA0092 | Co-expression vector for N-terminal His_6_-tagged Tse6 and Tsi6 from *P. aeruginosa* | This study |
| pETDuet-1::His_6_-PA0093_D11A::PA0092 | Co-expression vector for N-terminal His_6_-tagged Tse6^D11A^ and Tsi6 from *P. aeruginosa* | (Quentin et al., 2018) |
| pETDuet-1::His_6_-PA0093_H15A::PA0092 | Co-expression vector for N-terminal His_6_-tagged Tse6^H15A^ and Tsi6 from *P. aeruginosa* | This study |
| pETDuet-1::His_6_-PA0093_D11A_H15A::PA0092 | Co-expression vector for N-terminal His_6_-tagged Tse6^D11A, H15A^ and Tsi6 from *P. aeruginosa* | This study |
| pETDuet-1::FLAG-PA0091 | Expression vector for N-terminal FLAG tagged VgrG1 from *P. aeruginosa* | This study |
| pETDuet-1::PFL_6096_1-74-VSV-G::PFL_6095-His6 | Expression vector for C-terminal VSV-G tagged RhsANT and N-terminal His6-tagged EagR1 | This study |
| pRSETA::SL1344_0285 | Expression vector for SciW (for crystallization) | This study |
| pET29b::ECL_01566-VSV-G | Expression vector for C-terminal VSV-G tagged EagR_A_ from *E. cloacae* | This study |
| pET29b::SF0260a-VSV-G | Expression vector for C-terminal VSV-G tagged SF0260a (Eag) from *S. flexneri* | This study |
| pET29b:: SL1344_0285-VSV-G | Expression vector for C-terminal VSV-G tagged SciW from *S.* Typhimurium | (Quentin et al., 2018) |
| pET29b::PA0094-VSV-G | Expression vector for C-terminal VSV-G tagged EagT6 from *P. aeruginosa* | This study |
| pET29b::Spro_3016-VSV-G | Expression vector for C-terminal VSV-G tagged EagT6 from *S. proteamaculans* | This study |
| pET29b::PFL_6095-VSV-G | Expression vector for C-terminal VSV-G tagged EagR1 from *P. protegens* | This study |
| pET29b::PFL_6099-VSV-G | Expression vector for C-terminal VSV-G tagged EagT2 from *P. protegens* | This study |
| pET29b::FLAG-PFL_6094 | Expression vector for N-terminal FLAG tagged VgrG1 from *P. protegens* | This study |
| pET29b::PA0093_75-162-FLAG | Expression vector for C-terminal FLAG tagged PAAR domain of Tse6 | This study |
| pEXG2::ΔPFL_6095 | *eagR1* deletion construct | This study |
| pEXG2::ΔPFL_6099 | *eagT2* deletion construct | This study |
| pEXG2::ΔPFL_6209 | *tne2* deletion construct | This study |
| pEXG2::ΔPFL_6096 | *rhsA* deletion construct | This study |
| pEXG2::ΔPFL_6079 | *pppA* deletion construct | This study |
| pEXG2::ΔPFL_6096 ΔPFL_6097 | *rhsA*-*rhsI* effector-immunity pair deletion construct | This study |
| pEXG2::ΔPFL_6209 ΔPFL_6210 | *tne2*-*tni2* effector-immunity pair deletion construct | This study |
| pEXG2::ΔPFL_6094 | *vgrG1* deletion construct | This study |
| pEXG2::His_10_-PFL_6096 | N-terminal His_10_-*rhsA* fusion construct | This study |
| pEXG2::His_10_-PFL_6096* | N-terminal His_10_-*rhsA* fusion construct compatible with a strain lacking *eagR1* | This study |
| pEXG2::FLAG-PFL_6094 | N-terminal FLAG-*vgrG*1 fusion construct | This study |
| pEXG2::His_10_-PFL_6209 | N-terminal His_10_-*tne2* fusion construct | This study |
| pEXG2::PFL_6209-VSV-G | VSV-G | This study |
| pEXG2::PFL_6096_∆2-74 | RhsA NT deletion construct | This study |
| pEXG2::His_10_-PFL_6096_∆2-74 | RhsA NT deletion construct compatible in a strain with an N-terminal His_10_-*rhsA* fusion | This study |
| pEXG2::His_10_-PFL_6096_∆2-74* | RhsA NT deletion construct compatible in a strain with an N-terminal His_10_-*rhsA* fusion and lacking *eagR1* | This study |
| pEXG2::PA0093_D11A | Allelic exchange plasmid used to generate *tse6*^D11A^ in *P. aeruginosa* | This study |
| pEXG2::PA0093_H15A | Allelic exchange construct used to generate the *tse6*^H15A^ point mutation in *P. aeruginosa* | This study |
| pEXG2::PA0093_D11A_H15A | Allelic exchange plasmid used to generate *tse6*^D11,H15A^ in *P. aeruginosa* | This study |
| pEXG2::PA0093_∆16-61, ∆180-222 | Allelic exchange plasmid used to generate *tse6*^∆16-60,∆180-222^ in *P. aeruginosa* | This study |
| pSCrhaB2-V::PFL_6096_D1404A | Expression vector for RhsA^D1404A^ | This study |
| pSCrhaB2-V::PFL_6096_∆2-74_D1404A | Expression vector for RhsA_∆NT_^D1404A^ | This study |
